# PTSD and Burnout are Related to Lifetime Mood Spectrum in Emergency Healthcare Operator

**DOI:** 10.2174/1745017902016010165

**Published:** 2020-07-30

**Authors:** Claudia Carmassi, Carlo Antonio Bertelloni, Maria Teresa Avella, Ivan Cremone, Enrico Massimetti, Martina Corsi, Liliana Dell’Osso

**Affiliations:** 1 Department of Clinical and Experimental Medicine, University of Pisa, Pisa, Italy; 2 ASST, Bergamo Ovest, SSD Servizio Psichiatrico diagnosi e cura, Treviglio, Italy

**Keywords:** Trauma, Emergency, Health operators, Nurse, PTSD, Burnout, Depression, Mood

## Abstract

**Background:**

PTSD and burnout are frequent conditions among emergency healthcare personnel because exposed to repeated traumatic working experiences. Increasing evidence suggests high comorbidity between PTSD and mood symptoms, particularly depression, although the real nature of this relationship still remains unclear. The purpose of this study was to investigate the relationship between PTSD, burnout and lifetime mood spectrum, assessed by a specific scale, among health-care professionals of a major University Hospital in Italy.

**Methods:**

N=110 Emergency Unit workers of the Azienda Ospedaliero-Universitaria Pisana (Pisa, Italy) were assessed by the TALS-SR, MOODS-SR lifetime version and the ProQOL R-IV.

**Results:**

Approximately 60% of participants met at least one PTSD symptom criterion (criterion B, 63.4%; criterion C, 40.2%; criterion D 29.3%; criterion E, 26.8%), according to DSM-5 diagnosis. Almost sixteen percent of the sample reported a full symptomatic DSM-5 PTSD (work-related) diagnosis, and these showed significantly higher scores in all MOODS-SR depressive domains, as well as in the rhythmicity domain, compared with workers without PTSD. Further, mood-depressive and cognition-depressive MOODS-SR domains resulted to be predictive for PTSD. Significant correlations emerged between either PTSD diagnosis and criteria or ProQOL subscales and all the MOOD-SR domains.

**Conclusion:**

A significant association emerged among PTSD, burnout and lifetime MOOD Spectrum, particularly the depressive component, in emergency health care operators, suggesting this population should be considered at-risk and undergo regular screenings for depression and PTSD.

## 1 INTRODUCTION

Healthcare personnel are frequently and repeatedly exposed to stressful situations and traumatic events so they are more likely to develop pathological conditions such as burnout or Post-Traumatic Stress Disorder (PTSD), compared to the general population [1-3]. In light of these data, the last edition of the Diagnostic and Statistical Manual of Mental Disorders (DSM-5) significantly modified the diagnostic criteria related to the trauma for the PTSD diagnosis, specifying in Criterion A4 the impact of “experiencing repeated or extreme exposure to aversive details of the traumatic event(s)” addressing special populations such as emergency operators [4]. Previous studies reported PTSD rates up to ten-fold higher among emergency service workers with respect to the general population [5]. Particularly, PTSD rates range between 10% and 21% among medical doctors and nurses [6, 7]. A recent meta-analysis on 18 studies including over 30,000 subjects showed PTSD in 11% of ambulance personnel [8].

Several studies described also a frequent occurrence of burnout among health-care professionals, in particular among emergency unit operators with rates as high as 86% [2, 9]. Burnout of nurses and physicians in the emergency setting was favored by the stress of emotionally-charged decision-making as well as heavy workloads and extended work hours [10]. On one hand, burnout can affect physical and mental health, causing sleep pattern alteration, fatigue, concentration deficit and irritability; on the other hand, it can produce impairment in quality of care and patients’ health outcomes, besides increased absenteeism [11].

Considering the very high frequency of PTSD and burnout in emergency operators, and the detrimental impact of these syndromes not only on well-being but also on work-related functioning and patients outcome [12, 13], detecting individuals at risk is essential in order to develop effective prevention strategies. Different factors were found to predispose health workers to PTSD or burnout. From a socio-demographic perspective, being a young, female, nurse with low professional education levels are considered negative factors [2, 6, 14]. For what concerns organizational work factors, employee reward systems, peer and manager support, besides well-designed organizational structures improve resilience to stressful and traumatic events experienced on duties [15]. Moreover, some operator’s psychological characteristics or pre-existing psychiatric disorders are related to a higher risk. In this regard, a great amount of literature shows a strict but controversial relationship between mood disorders, particularly depression, and trauma and stress-related disorders [16-18].

PTSD and depressive symptoms show a high tendency to co-occur after a traumatic event [19, 20] and in epidemiological samples about 50% of subjects with PTSD also reported a depressive disorder [18, 21, 22]. According to a French study based on a large population sample, one-third of patients with moderately PTSD showed comorbid depressive symptoms. This percentage was higher in those with severe PTSD, who presented comorbid depression in more than half of cases [23]. Furthermore, another study reported higher comorbidity rates between PTSD and Major Depressive Disorder (84.4%) or Dysthimia (41.9%). In particular, female gender and childhood sexual or physical abuse were positively associated with depressive comorbidity in patients with PTSD [24]. For what concern emergency personnel, the prevalence of PTSD, depression, and high general psychological distress was higher among medical workers (6.6%, 14.3%, and 14.5%, respectively) than among other professional categories such as firefighters (1.6%, 3.8%, and 2.6%, respectively) [25]. On the whole, PTSD-depression comorbidity is usually associated with a higher functional impairment than a single disorder [26].

These high comorbidity rates have various explanations. According to the causality model, PTSD is a causal risk factor for the onset of depressive disorders. Prospective studies on large Veteran samples found that PTSD diagnosis at the basal evaluation was a predictive factor for depression in a 24-month follow-up [27]. Data on civilian sample corroborate the hypothesis that post-traumatic stress symptomatology could be a risk factor for the onset of depression [16, 21, 28, 29]. In contrast, other authors suggested that pre-existing depression could predispose to PTSD after a traumatic event [30-35]. In a study on 6,744 male twins from the Vietnam Era Twin Registry, the risk of developing PTSD after trauma exposure was increased in those with pre-existing depressive or anxiety disorders [30]. Similarly, Shiller-Allon *et al.*, reported that depressive symptoms were related to an increase in post-traumatic distress in a sample of 156 Israeli trauma victims followed-up for 12 weeks after being exposed to trauma [31].

Exploring, upon *Mood Spectrum Model* approach, the relationship between lifetime mood spectrum symptoms and PTSD, some of us reported manic and depressive lifetime symptoms to be related to the severity of post-traumatic stress symptomatology and suicidality [32-35]. These findings support the hypothesis that depressive symptoms may predispose to PTSD after the exposure of a traumatic event.

A positive correlation between burnout and depression represents a corroborated finding [36, 37]. Three longitudinal studies specifically described a direct link from depression to burnout [38-40]. In a long longitudinal study on 297 university students, Salmela-Aro *et al.* found that participants with a higher depression trajectory showed higher burnout levels in the long period with respect to those with lower depression, suggesting that depression may be a risk factor for burnout [40].

Considering the scant data about the role of depressive symptoms as vulnerability factors of post-traumatic stress reactions among emergency personnel, the purpose of this study was to evaluate lifetime mood spectrum symptoms in emergency personnel and their association with PTSD and burnout in order to identify a subject at risk for developing these conditions. To specifically investigating affective symptoms during the lifespan of the participants, a *mood spectrum model* was adopted for this study. There is growing evidence that mood disorders should be regarded as a spectrum of manifestations, rather than categorical disorders. A *mood spectrum model* is a dimensional approach that evaluates not only unipolar and bipolar symptoms, but also sub-threshold manifestations, atypical symptoms and behavioral traits, in the light of a lifetime perspective [41]. This approach was found to be a valid method to better define mood disorders in terms of severity, prognosis, treatment implications and relationship with other symptoms [41-43].

## 2 MATERIALS AND METHODS

### 2.1 Study Sample

The study sample included 95 emergency unit workers of the “Azienda Ospedaliero-Universitaria Pisana (AOUP)”, Italy. The participants' enrollment was conducted between September 2013 to March 2014. Full data were available only for 82 participants from the whole sample because the remaining 13 ones (2 males and 11 females; 3 medical doctors, 5 nurses and 5 healthcare assistants) gave partial responses.

The study was conducted in accordance with the Declaration of Helsinki. The ethics committee of the University of Pisa, Italy, approved all the procedures of evaluation and recruitment. Suitable candidates provided written informed consent after receiving an exhaustive description of the study and after having the opportunity to ask any questions in reference to the study. Participants completed the questionnaires at the end of the work-shift in a stand-alone room to ensure their privacy.

### 2.2 Diagnostic Instruments

Participants were investigated using the Trauma and Loss Spectrum - Self Report (TALS-SR), to investigate PTSD and posttraumatic stress spectrum symptoms related to the work activity [44, 45], the MOOD Spectrum – Self Report, Lifetime version (MOODS-SR), in order to explore lifetime mood symptomatology [41] and the Professional Quality of Life Scale - Revision IV (ProQOL R-IV) to examine compassion satisfaction, burn-out and compassion fatigue related to work activities [46].

The TALS-SR is a questionnaire developed for assessing posttraumatic stress spectrum symptoms. It includes 116 items exploring the lifetime experience of a range of losses and/or traumatic events and lifetime symptoms, behaviors, and personal characteristics that might represent manifestations and/or risk factors for the development of a stress response syndrome. The instrument is organized into nine domains including *loss events* (I), *grief reactions* (II), *potentially traumatic events* (III), *reactions to losses or upsetting events* (IV), *re-experiencing* (V), *avoidance and numbing* (VI), *maladaptive coping* (VII), *arousal* (VIII), and *personal characteristics/ risk factors* (IX). The responses to the items are coded in a dichotomous way (yes/no), and domain scores are obtained by counting the number of positive answers.

Due to the sample characteristics, criterion A was considered satisfied. Particularly, the instrument was adapted, in accordance with the aim of the present study, to assess symptoms due to traumatic experience related to the work as emergency personnel (criterion A4).Consequently, only the following TALS-.Sr domains were included in the study: *reactions to losses or upsetting events* (IV), *re-experiencing* (V), *avoidance and numbing* (VI), *maladaptive coping* (VII), *arousal* (VIII). All participants were asked to report symptoms related to work-related trauma exposure. According to previous studies [47, 48] a DSM-5 diagnosis of PTSD was assessed by using the following matching between DSM-5 symptoms criteria and TALS-SR items:

- criterion B (B1 =80; B2 =77; B3 =79; B4 =78; B5 =81);
- criterion C (C1 =86; C2 =87 and/or 88 and/or 89);
- criterion D (D1 =90; D2 =95; D3 =85; D4 =96; D5 =91; D6 =93; D7 =92); and
- criterion E (E1 =108; E2 =99 and/or 100 and/or 102; and/or 103 and/or 104; E3 =106; E4 =107; E5 =105; E6 =109).

The TALS-SR presented good intra-class correlation coefficients (from 0.934 to 0.994) with SCI-TALS; the interview version was used for assessing post-traumatic stress symptomatology. Similarly, SCI-TALS showed a good internal consistency (Kuder-Richardson coefficient exceeding the minimum standard of 0.50 for each domain).

The MOODS-SR is a 161-item questionnaire coded as present or absent for one or more periods of at least 3-5 days throughout the subject’s lifespan. Not only *manic* and *depressive components* are investigated, but also disturbances in *rhythmicity and vegetative functions* are assessed. Both the manic and the depressive components are subtyped into three domains exploring *mood*, *energy* and *cognition* symptoms respectively. The number of the *mood*-, *energy*- and *cognition-manic* items endorsed by subjects makes up the *manic component* (62 items), while the sum of the *mood*-, *energy*- and *cognition-depressive* items constitutes the *depressive component* (63 items). The *rhythmicity and vegetative functions* domain (29 items) explore alterations in the circadian rhythms and vegetative functions, including changes in energy, physical well-being, mental and physical efficiency related to the weather and season, and changes in appetite, sleep and sexual activities. The questionnaire showed good internal consistency, with Kuder-Richardson's coefficient ranging from 0.79 to0.92 among single domains.

The ProQOL is a 22 item self-report measure to assess three different dimensions: *compassion satisfaction*, *burnout* and *compassion fatigue* related to work. Respondents were asked to indicate how often (from 0-never to 5-very often) during the last 30 days, each item was experienced [47-49]. The *compassion satisfaction* measures pleasure derived from being able to do your work well, whereas high scores represent a greater satisfaction related to your ability to be an effective caregiver. The *burnout* dimension in this scale is associated with feelings of hopelessness and difficulties in dealing with your work. The *compassion fatigue* dimension is about work-related secondary exposure to stressful events. High scores indicate that you are exposed to frightening experiences at work. Cronbach’s alpha values of the three subscales were 0.80 for CF, 0.89 for CS, and 0.71 for BO. The Italian version of the instrument was validated.

### 2.3 Statistical Analysis

All statistical analyses were carried out using the Statistical Package for Social Science, version 23.0 (SPSS Inc., Chicago 2018). The descriptive procedures were used to evaluate the demographic characteristics of the sample as well as to calculate the frequency of the PTSD and the scores of the TALS-SR, MOODS-SR and ProQOL domains. Considering that MOODS-SR domain scores were not normally distributed, the non-parametric Man-Whitney test was computed in order to compare MOODS-SR domains between subjects with PTSD and without PTSD. A logistic regression model was computed to examine the role of the MOODS-SR domains, education level and gender as a predictive variable of PTSD diagnosis, considered as the dependent variable. Spearman's correlation coefficients were calculated to investigate possible associations between the TALS-SR domains or ProQOL dimensions and the MOODS-SR domains.

## 3 RESULTS

Most of the participants were nurses (55, 67.1%), followed by health-care assistants (14, 17%) and medical doctors (13, 15.6%, including physicians and residents). Regarding socio-demographic features, 50 (61%) were females and 32 (39%) were males; the mean age was 40.34±8.10 (min 25, max 61), and 59 (72%) were graduated (bachelor’s or advanced degree).

In the sample, 13 (15.9%) participants were found to be affected by DSM-5 PTSD, while higher percentages of each PTSD criterion were reported: criterion B (re-experiencing) was presented by 50 (63.4%) subjects; criterion C (avoidance) by 33 (40.2%); criterion D (alteration in mood and cognition) by 24 (29.3%); and finally, criterion E (Hyperarousal) by 22 (26.8%) subjects. According to the ProQOL subscales, the mean score of the *compassion satisfaction* was 31.60±5.0, the *burnout* one was 14.89±4.92 and the *compassion fatigue* one was 10.84±4.92. The mean scores of the MOODS-SR depressive domain scores were respectively 4.61±4.59, 1.29±1.64 and 2.73±3.73 for *mood-depressive*, *energy-depressive* and *cognition-depressive*. The *depressive component* score was 8.63±8.66. On the other hand, the scores of the *mood-manic*, *energy-manic* and *cognition-manic* MOODS-SR domain were 6.28±5.12, 2.18±2.38 and 3.34±3.63 respectively, whereas the *manic component* score was 11.80±10.02. Finally, the *rhythmicity* domain mean score was 5.94±5.21.

Subjects with PTSD reported significantly higher scores in the *mood-depressive* (3.59±3.77 *versus* 10.00±4.90, p<.001), *energy-depressive* (1.12±1.51 *versus* 2.23±2.05, p=.025) *cognition-depressive* (1.83±2.45 *versus* 7.54±5.47, p=.001) and *rhythmicity* (5.1±4.29 *versus* 10.23±7.41, p=.016) domains, besides in the *depressive component* (6.54±6.70 *versus* 19.77±9.56 4.33 p=<.001). (Table **1**)

In a logistic regression model (sensitivity=53.8% and specificity=95.6%.), considering the gender and education, besides MOODS-SR *mood-depressive*, *energy-depressive*, *cognition-depressive* and *rhythmicity* scores as independent variables, and the PTSD diagnosis as the dependent variables, the *mood-depressive* [b=0.256 (SE=0.108), p=.018] and the *cognition-depressive* [b=0.268 (SE=0.121), p=.026] domains scores presented a significant positive association with the PTSD (Table **2**). The ROC curve and the characteristics of the regression model are shown in Fig. (**1** and Table **2a**), respectively.

Finally, significant correlations emerged between DSM-5 PTSD diagnosis and criteria, or ProQOL subscales and MOOD-SR domains (Table **3**). Particularly, significant positive moderate correlations emerged between the *mood-depressive* MOODS-SR domain and the total PTSD symptoms scores (r=.513) the cognitive and mood alterations criterion symptoms (r=.519) and the hyperarousal criterion symptoms (r=.517); between the *cognition-depressive* MOODS-SR domain and the total PTSD symptoms scores (r=.563) and the cognitive and mood alterations criterion symptoms (r=.560). Furthermore, the MOODS-SR mood-depressive domain showed a significant low positive correlation with ProQOL burnout (r=376) and *compassion fatigue* (r=.338).

## 4 DISCUSSION

To the best of our knowledge, this is the first study that aimed at exploring the relationship between lifetime mood spectrum symptoms and PTSD or burnout among emergency Healthcare operators in Italy. Almost 16% of the sample reported symptomatic PTSD due to trauma related to healthcare emergency work. This result is in line with previous studies and meta-analysis on a similar sample [5, 6, 49, 50] and corroborates the data about high risk for trauma-related psychopathology in emergency workers. Thus, specific training and systematic PTSD prevention program are needed for emergency operators in order to improve their mental health and quality of life. The subjects filled the evaluation in a stand-alone room, after the work-shift in order to ensure their privacy

Furthermore, PTSD diagnosis was found to be associated with lifetime mood spectrum symptoms, especially with the depressive ones. In the National Comorbidity Survey-Replication, more than half of the subjects affected by PTSD presented a lifetime diagnosis of Major Depressive Disorder [51]. The relationship between post-traumatic stress reactions and depression is considered complex, with overlapping symptomatology, mutual influence, and very high comorbidity rates [16, 19, 24, 52]. In the last years, the new broad approach to PTSD proposed by DSM-5, encompassing the negative alterations in cognition and mood symptoms, such as guilt, anger or shame feelings, enhanced the conceptual link between these two disorders [4].

Despite most of the existing studies are focused on the role of trauma or PTSD as a risk factor for depressive symptoms [28, 29, 53], some data also showed an inverse trend [30-35, 54, 55]. Some authors, in fact, showed how some features related to a depressive state, such as an impairment of coping skills and greater stress perception could lead to vulnerability to traumatic events [56]. Negative self-evaluation and reduced initiative may determine cognitive and behavioral avoidance in dealing with trauma-related stimuli, compromising fear extinction [57]. Moreover, high neuroticism and low extroversion represent underlying personality risk factors shared between depression and PTSD [16]. On one hand, high levels of neuroticism facilitate reaction to stressors and frustrations by means of negative affect; on the other hand, low levels of extroversion reduce the tendency to seek support from others in order to face upsetting events. All these elements could be considered as impairment in the self-efficacy belief that is crucial for the recovery of a traumatic experience [31].

The results of the present study suggested how the vulnerability produced by lifetime depressive spectrum symptoms could be particularly relevant in the professional population, like emergency operators, exposed every day to a traumatic experience or stressful situations. In this chronic context, the impairment in coping with traumas due to low self-esteem, negative affectivity or cognitive deficit is a likely high risky condition to PTSD development and maintenance. A recent study conducted in Pakistan, on over 500 emergency medical personnel, indicated depression as a predictive factor for work-related PTSD [58]. Furthermore, in a German survey on a large sample of emergency physician and paramedic staff conducted with the purpose to explore post-traumatic stress symptoms and depression rates, the authors recommended that those who presented post-traumatic stress symptoms should be investigated for depression and vice versa, considering the strong correlation between these two dimensions [59].

The logistic regression model showed that mood and cognitive lifetime depressive symptoms are associated with PTSD diagnosis. These results appear to be in line with the decision of the DSM-5 to introduce the new diagnostic criterion D (negative alteration in mood and cognition) for PTSD diagnosis, highlighting the importance of negative emotions and distorted cognition as a manifestation of the clinical picture of the disorder [4]. In this regard, in the Spearman analysis, the strongest correlations emerged between mood-depressive and cognition-depressive MOODS-SR domains and the PTSD diagnosis, as well as the PTSD criteria re-experiencing (B), alteration in mood and cognition (D) and Hyperarousal (E). It may be possible that the tendency to low self-esteem, negative feelings like sadness, guilt or shame, anhedonia and other affective or cognitive depressive spectrum symptoms is exacerbated by trauma exposure and evolved in PTSD. Some authors reported a dimensional commonality between depression and some PTSD symptoms, such as detachment, restricted affect, loss of interest, sense of foreshortened future, irritability, and cognitive impairment [60]. Furthermore, negative emotions like guilt and shame feelings were related to the onset of PTSD and its severity [61, 62].

Significant associations also emerged for the burnout and the compassion fatigue subscale of the ProQOL. Several studies showed circular influences between burnout and depression: if on one hand burnout has been hypothesized to represent an initial phase in depression, on the other hand, this latter may negatively influence the experience of work-related stress, leading to burnout [37, 63]. The nature of this relationship seems to be very similar to that emerged between depression and PTSD.

It is noteworthy that a significant association with PTSD clusters also emerged for the rhythmicity MOODS-SR domain. This result appears to be in line with existing data about the role of altered vegetative functions and biological rhythms in PTSD [64]. Sleep disturbances, somatic complaints, impairment in sexual or eating behaviors, as well as seasonal/circadian rhythms alterations were found not only to be associated with post-traumatic stress symptoms [64-69], but also to suicidality in PTSD patients [64].

This study has several limitations. The first one is the limited sample size and lack of a control group that may affect results. Furthermore, full data were not available from 13 enrolled subjects that were excluded from the study. Secondly, although TALS-SR has been used to assesses symptomatic PTSD according to DSM-5 criteria across studies [47, 48], the use of a self-report instrument may be considered less reliable and more influenced by co-occurring events than a standardized structured interview. Thirdly, a selection bias may be present, as subjects with PTSD, showing severe avoidant symptoms, may have not been enrolled. Fourthly, the lack of full evaluation for the whole sample. Lastly, participants were not screened for Axis I psychiatric comorbidities, which may impact significantly on work functioning.

## CONCLUSION

Despite the limitations mentioned above, this study showed a relevant association between lifetime depressive symptoms and burnout, as well as work-related PTSD in emergency health operators. It is conceivable that this at-risk population should undergo regular screenings for depression and PTSD in order to better manage the symptoms due to stress and trauma exposure during work. We may argue that this issue is even more relevant in the framework of the Covid-19 emergency, and its possible impact on the mental health of the healthcare operators. Detecting specific risk factors for this population could be useful to assess vulnerable subjects and consequently prevent post-traumatic stress sequelae.

The present study corroborates previous findings on the complex relationship between “mood” and trauma-related psychopathology, and especially highlights it in health emergency operators. Further longitudinal research studies are required to understand the impact of trauma on this population in order to potentiate prevention interventions and therapeutic strategies.

## Figures and Tables

**Fig. (1):**
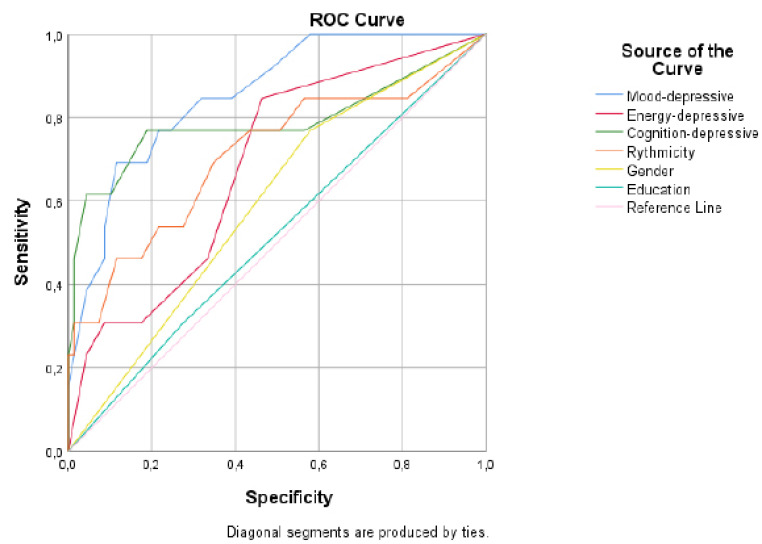
ROC curve of the regression model.

**Table 1:** Comparison of the MOODS-SR domains scores between healthcare operators without PTSD (N=69) and those with PTSD (N=13).

	**No-PTSD** **Mean ± SD**	**PTSD** **Mean ± SD**	**Cohen’s** **d**	**Mann-Whitney** **z**	**p**
Mood-depressive	3.59±3.77	10.00±4.90	1.47	4.07	**<.001**
Energy-depressive	1.12±1.51	2.23±2.05	0.62	2.24	**.025**
Cognition-depressive	1.83±2.45	7.54±5.47	1.35	3.42	**.001**
Depressive component	6.54±6.70	19.77±9.56	1.60	4.33	**<.001**
Mood-manic	5.99±5.11	7.85±5.11	0.36	1.23	.218
Energy-manic	1.97±2.21	3.31±2.97	0.51	1.68	.093
Cognition-manic	3.03±3.52	5.00±3.89	0.53	1.93	.054
Manic component	10.99±9.70	16.15±10.10	0.52	1.68	.093
Rhythmicity	5.1±4.29	10.23±7.41	0.85	2.41	**.016**

**Table 2:** Logistic regression model: MOODS-SR depressive and rhythmicity domains, gender and education levels as predictive variables associated with PTSD diagnosis in the total sample.

**Predictive factors**	***B(S.E.)***	***O.R.***	***CI95%***	***p***
Mood-depressive	0.256 (0.108)	**1.292**	1.045-1.597	**.018**
Energy-depressive	-0.309 (0.275)	0.734	0.429-1.258	.261
Cognition-depressive	0.268 (0.121)	**1.308**	1.032-1.657	**.026**
Rhythmicity	0.051 (0.102)	1.052	0.861-1.286	.618
Gender	0.548 (1.024)	1.731	0.232-12.885	.592
Education	1.048 (0.967)	2.851	0.428-18.892	.279
K	-6.671 (2.237)	0.001	-	.003

Cox R^2^ = 0.31; Nagelkerke R^2^ = 0.52; Hosmer-Lemeshow test: χ^2^ = 7,24, p = .511; Global-goodness-fit percentage = 89,0%. Sensitivity=53.8%; specificity=95.6%.

**Table 2a:** ROC curve characteristics of the regression model.

**Predictive Factors**	***Area (S.E.)***	***CI95%***	***p***
Mood-depressive	.855 (.053)	.752-.959	.000
Energy-depressive	.685 (.076)	.535-.835	.035
Cognition-depressive	.790 (.091)	.611-.968	.001
Rhythmicity	.711 (.090)	.535-.887	.016
Gender	.595 (.082)	.434-.756	.281
Education	.516 (.089)	.343.690	.854

**Table 3:** Correlations (r) between MOODS-SR depressive domains and PTSD symptoms clusters or ProQOLsubscales.

-	**Mood Depressive**	**Energy Depressive**	**Cognition Depressive**	**Rhythmicity**
PTSD and PTSDdiagnostic criteria				
Total PTSD symptoms	.513**	.249*	.563**	.360*
B) Re-experiencing symptoms	.429**	.353*	.328*	.304*
C) Avoidance symptoms	.343*	.188	.335*	.245*
D) Cognitive and mood alterations symptoms	.549**	.360*	.560**	.437**
E) Hyperarousal symptoms	.517**	.380*	.437**	.316*
ProQOL Subscale				
Compassion Satisfaction	-.041	.052	.075	.047
Burnout	.376*	.087	.281*	.289*
Compassion Fatigue	.338*	.152	.193	.150

Moderate positive correlations (.5 > r < .7); Low positive correlations (.3 > r < .5); *p<.05;**p<.001"

## LIST OF ABBREVIATIONS

PTSD: = Post-Traumatic Stress Disorder
DSM-5: = Diagnostic and Statistical Manual of Mental Disorders
